# Effectiveness of a community-based intervention (Konga model) to address the factors contributing to viral load suppression among children living with HIV in Tanzania: a cluster-randomized clinical trial protocol

**DOI:** 10.1093/biomethods/bpac002

**Published:** 2022-01-07

**Authors:** Mageda Kihulya, Leornard K Katalambula, Ntuli A Kapologwe, Pammla Petrucka

**Affiliations:** 1 School of Nursing and Public Health, University of Dodoma, PO Box 395, Dodoma 41210, Tanzania; 2 President’s Office—Regional Administration and Local Government, PO Box 1923, Dodoma 41207, Tanzania; 3 Research & Graduate Studies, College of Nursing, University of Saskatchewan, 4400-4th Avenue, Regina, SK S4T 0H8, Saskatoon, Canada

**Keywords:** viral-load suppression, antiretroviral therapy, HIV-infected children, community-based intervention, Tanzania

## Abstract

This study aims to test the effectiveness of a community-based intervention (Konga model) to improve viral-load suppression in children living with human immunodeficiency virus (HIV) and enrolled in care and treatment centers in Tanzania mainland. The study will be a cluster-randomized clinical trial study designed with both intervention and control arms. The study will involve 268 children with a viral load of >1000 copies/ml who are aged between 2 and 14 years. The children will be randomly allocated into the intervention and control arms. The intervention will include three distinct activities: adherence and retention counseling, psychosocial support, and comorbidity screening (i.e. tuberculosis). The outcome of the study will be assessment of the success of the intervention to increase medication adherence with the immediate result of reducing the viral load below 1000 copies/ml. Descriptive statistics will be used to calculate the mean, median, standard deviation, and interquartile range of continuous data. We will use frequencies and percentages to summarize categorical data. As for the primary outcome (proportion of HIV-infected children with viral suppression), we will compare the proportion of successful participants in the intervention and control arms. Proportions and tests for different proportions will be used as a measure of improvement. All statistical tests will be two-sided and *P* < 0.05 will be considered statistically significant.

## Introduction

The retrovirus human immunodeficiency virus (HIV) attacks the body’s cellular immune system and acquired immunodeficiency syndrome (AIDS) can eventually result [1]. The attack depletes CD4 cells, which leaves people vulnerable to illnesses that a healthy immune system would otherwise eliminate [2–4]. Retroviral infection remains the leading cause of morbidity and mortality throughout the world [5]. However, with the introduction to antiretroviral therapy (ART) and wide accessibility, mortality has been significantly reduced [6–8]. Thus, HIV infection has become a manageable chronic health condition, enabling people living with HIV (PLHIV) to live long and quality healthy lives [9].

Viral-load measurement emerged as an essential monitor of a therapy’s effectiveness after ART initiation and it is considered a surrogate marker for disease progression [3, 10]. ART in children aims to suppress HIV replication and halt disease progression while reducing opportunistic infections and morbidities [11, 12].

Viral-load suppression (VLS) after early ART initiation is the primary goal in children [13]. Globally, about 400,000 children living with HIV under 15 years of age who are receiving ART and living in low- and middle-income countries have not achieved VLS [14]. In East-African nations, compared with other countries in sub-Saharan Africa, a low proportion of children on ART achieved VLS [15, 16]. Thus, there is high risk of developing AIDS. In Tanzania, program data and the Tanzania HIV Impact Survey (THIS) have demonstrated that VLS in pediatric patients continues to be low [4, 17, 18].

Several studies have cited factors causing unsuppressed viral loads, such as poor adherence and co-morbidities [14, 19, 20], malnutrition [21, 22], and underlying tuberculosis (TB) infection [23]. These patients who initially received ART still have advanced disease [24].

Despite substantial ART coverage in other groups living with HIV in Tanzania, the VLS among HIV-positive children on ART remains unacceptably low at 18%. That means that 82% of those enrolled in care and treatment centers (CTCs) and receiving ART did not achieve VLS [4]. Several studies have demonstrated that early enrollment of children to ART and maintaining good adherence (retention) reduces HIV replication and suppresses the virus [25, 26]. Through the National HIV/AIDS Control Program, the Government of Tanzania has made efforts to improve retention and adherence for PLHIV. These efforts have not however been effective for VLS among children due to the loss of follow-up of caregivers and their HIV-exposed infants [27].

Trials in different countries have demonstrated that community-based intervention improves HIV care services among children living with HIV and AIDS [25, 28–30]. Thus, UNAIDS [31] recommends a requirement of sustained engagement and unique inputs from various communities, from small informal groups at the grassroots level up to global coalitions.

We therefore identified the need for a sustainable intervention that promotes retention and adherence to ART and that addresses low VLS among children living with HIV in Tanzania.

## Material and methods

### Study area

We will conduct this study in the Simiyu Region. Administratively, the region is comprised of six district councils with a total of 218 health facilities (8 hospitals, 17 health centers, and 193 dispensaries), of which 106 sites provide ART.

### Study design and population

We will use a cluster-randomized trial study designed with both intervention and control arms. The study will involve 268 children (134 controls and 134 patients who receive intervention) aged 2–14 years who are attending CTC and who have a viral load of >1000 copies/ml.

### Recruitment

The healthcare workers (i.e. ART nurses) will identify and recruit children with a viral load of >1000 copies/ml and who were aged 2–14 years. Before recruitment, informed consent will be obtained from their caregivers.

### Intervention

The UNAIDS and previous studies have recommended the engagement of communities, from small informal groups at the grassroots level up to global coalitions, to improve HIV care [31]. The National Council of PLHIV (NACOPHA) is a non-profit, non-government organization. It is a national grassroots-based organization of all individuals who are recognized through organized groups and clusters of PLHIV in Tanzania mainland. Since its establishment has embarked upon coordinating the efforts of PLHIV through their district clusters known as “Konga” to address the needs of PLHIV. Konga is a Swahili name meaning that is a Cluster of PLHIV at the district level, thus Konga is the smallest unit of NACOPHA. Hence, we will use the community of PLHIV known as Konga to provide services to children.

### Plan for the intervention

#### Role of the health worker

The health workers, usually the ART nurses at the selected facility, will identify children with low VLS and link them to the Konga. They will also continue routine care of the child.

#### Roles of the Konga

We will provide the standard operating procedures (SOPs) to the Kong such that different person will use for the provision of the desired intervention. The SOP will have specific standards adapted from the National HIV care and treatment guidelines, thus the Konga will use the SOP:

- To provide enhanced adherence counseling and intensive follow-up. Here, the Konga will routinely visit the client’s house and at every visit they will assess and document:
  ∘ Medication regimen, storage of drugs at home.
  ∘ Doses missed: how often, why, then solve problem with the client.
  ∘ Side effects such as headache, nausea and vomiting, diarrhea, fatigue, sleeping, difficulty, a dry mouth, a rash, dizziness and a pain and client response.
  ∘ Use of other medications, including traditional medicines.
  ∘ Challenges.
- Follow-up screening for TB and other comorbidities (members of the Konga will visit the children and screen for opportunistic infection and encourage them to visit the health facility for further management); and
- Provision of psychosocial support by members of the Konga team.

### Outcome measures

The study will have two outcome measures. The primary measure will be the adherence, this will be measured by using the standard adherence tool (National AIDS Control Program [32]) and loss to follow-up (retention in CTC), which will be compared in both arms. We will measure these metrics at baseline, subsequent follow-up, and the study’s end (i.e. 6 months). The secondary measure will be the viral load, which will be measured at the baseline and subsequently at the 6-month study’s end, comparing both arms.

### Sample size calculation and selection

The trial will be conducted in 20 CTC clinics that deliver HIV care and treatment within the four selected districts (10 CTCs will carry out the intervention and 10 CTCs will carry out standard care). We determined the study size from the primary outcome of proportional HIV-positive children with a viral load >1000 copies/ml. According to THIS (2018), 18% of HIV-positive children under the age of 15 years had a viral load >1000 copies/ml; thus, we assumed that value for standard care. Therefore, under a cluster design, the study will need a total sample size of about 268 children (134 in the intervention group and 134 in the control group) to increase the proportion of children with viral suppression from 18% to 30% with 80% power, ata5% significance level, an intra-cluster correlation of 0.01, and 10% expected loss to follow-up. We carried out this power calculation using the Stata™ function “clustersampsi” command (clustersampsi, binomial p1[0.18] p2[0.3]k[20] rho[0.01] alpha[0.05] beta[0.8]).

### Selection and randomization of CTC

**Figure 1:**
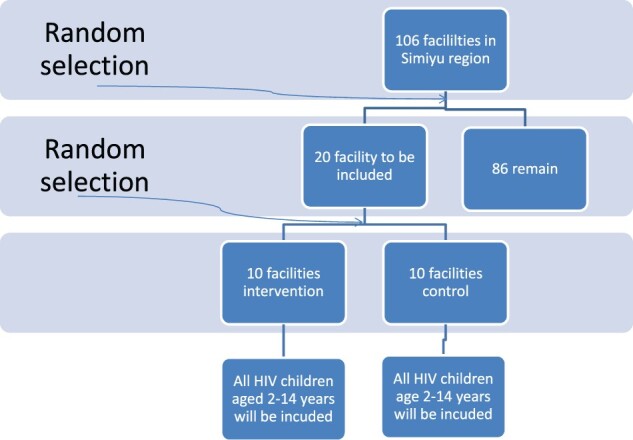
study design and flow chart.

A site assessment will be performed before the final site selection and sites will be selected to participate in the study based on infrastructure feasibility, last numbers of children enrolled into CTC during the past year, numbers and qualification of staff affiliated to CTC and balanced representation of the different level of health facilities and urban versus rural affiliations. Assignment of sites to one of the two study arms will be done by randomization (Fig. 1). Stratification during the randomization process (e.g. rural versus urban) will be considered.

The study will be open-label whereby the Konga, caretakers, and the children will be aware of the intervention received since neither the outcome assessor nor person receiving the intervention will influence the outcome. The study’s primary outcome is to reduce the viral load <1000 copies/ml.

### Data collection

#### Baseline data

At the beginning of the study, we will collect baseline data using a checklist. We will collect information from caregivers and their HIV-affected children. From the caregiver, we will collect data, such as social demographic characteristics, age and sex, level of education, income, and marital status. We will collect data, such as age, sex, and clinical characteristics of the children, and also weight, CD4 count, and VLS. Other data collected from the child will include anthropometric measurements, nutrition status, medication adherence, and opportunistic infection status.

#### Follow-up data

We will follow participants monthly in both the intervention and control arms and observe adherence, retention, and opportunistic infections.

#### End of study (6 months)

At the 6-month point, we will collect data from both arms regarding the viral load, weight, and number of missing doses (adherence).

### Measurement of variables

#### Dependent variable

The dependent variable will be the viral load cell count and it will be classified as suppression when the count is <1000 copies/ml and unsuppressed when >1000 copies/ml.

#### Independent variable

The independent variables will be age, weight, adherence, and opportunistic infection (i.e. TB). The caregivers’ social demographic characteristics, age, sex, level of education, income, and marital status will be the independent variables.

### Data processing and analysis

We will enter data into Excel™ software and analyze it using Stata™ software. Descriptive statistics will summarize continuous data, such as the mean, median, standard deviation, and interquartile range. We will use frequencies and percentages to summarize categorical data. Multiple statistical tests and methods will be employed. The univariate analysis will typically utilize Pearson’s chi-squared correlation or Fisher’s exact test, where appropriate. As for the primary outcome (mean difference in the viral load of HIV-positive children with VLS), we will use an independent *t*-test to compare the means of participants in the intervention and control arms. We will develop a multivariable regression model, adjusting for clustering and other confounders for potential confounding. We will use proportions and tests for different proportions as measures of improvement using the difference in difference. All statistical tests will be two-sided and *P* < 0.05 will be considered statistically significant.

### Dissemination of results

We will present the findings from this study to the Simiyu Regional Health Management Team. We will also present this study at the President’s Office, Regional Administrative Secretary, and Local Government’s (PO-RALG) Directorate of Health, Nutrition, and Social Welfare. We will present the finding at local and international conferences and publish the study in an international peer-reviewed journal.

### Ethical clearance and consent to participate

In the first stage, we will obtain the ethical clearance and registration for this study from the Dodoma University Ethical Board. In the second stage, we will require permission to use the CTC2 database from the Ministry of Health. We will seek consent from other authorities in the third phase including the PO-RALG. In the fourth stage, we will seek permission from the person in-charge of health facilities. In the last step, we will seek approval and written consent from participants. Our study was registered in the Pan African Clinical Trial Registry with a registration number PACTR202111867711522.

## Discussion

This Konga model study will be the first cluster-randomized clinical control trial study in Tanzania using the community as a basis for addressing challenges in VLS. The existing community of PLHIV will enhance ART adherence with VLS in children.

A systematic review of community-based interventions highlighted the importance of carefully designing organization-based HIV prevention interventions in a way that would improve their effectiveness and efficiency [33]. The current study will use community participation to promote retention and adherence to treatment, focusing on home-based follow-up and psychosocial and peer support [29]. Several studies have demonstrated that poor adherence and retention in ART care detract from VLS among children [34–37] living with HIV. In this intervention, we will use the Kong to promote retention and adherence to ART among children receiving ART to reduce their viral loads.

Opportunistic infections, such as TB, have been shown to hamper VLS in children receiving ART [14, 19, 20, 38]. Another study demonstrated that the initiation of ART in children reduces the incidence of TB [24]. The Konga model will include active home visits to screen children for TB and other co-morbidities in the control arm. The screening will help to identify early infection [24] and facilitate referral to the treatment point.

HIV-infected children potentially suffer from mental-health disorders that result in poor quality of life, HIV disease progression; poor compliance and increased mortality [39]. Studies have demonstrated that psychiatric morbidity in HIV-infected children is higher than that in children in the general population. These studies recommended that there is a need to incorporate psychiatric liaison service routine care for HIV-infected children [40–42]. The personnel from the Konga will visit the children and their families to provide psychosocial counseling and support.

## Data availability

Not applicable because this is a protocol manuscript which contain no any data.
